# 6,8-Di­bromo-4-oxo-4*H*-chromene-3-carbaldehyde

**DOI:** 10.1107/S1600536814005327

**Published:** 2014-03-15

**Authors:** Yoshinobu Ishikawa

**Affiliations:** a School of Pharmaceutical Sciences, University of Shizuoka, 52-1 Yada, Suruga-ku, Shizuoka 422-8526, Japan

## Abstract

In the title compound, C_10_H_4_Br_2_O_3_, the atoms of the 6,8-di­bromo­chromone unit are essentially coplanar [largest deviation from the mean planes = 0.1109 (3) Å] and the formyl group is twisted slightly with respect to the attached ring [C—C—C—O torsion angles = 11.5 (4) and −168.9 (3)°]. In the crystal, mol­ecules are linked to each other through halogen bonds [Br⋯O = 3.118 (2) Å, C—Br⋯O = 162.37 (8) and C=O⋯Br = 140.20 (15)°]. The molecules are further assembled *via* π–π stacking interactions [centroid–centroid distance = 3.850 (2) Å].

## Related literature   

For the biological activity of the title compound, see: Kawase *et al.* (2007 ▶). For its use as a starting material for the synthesis of alkaline phosphatase inhibitorsrelated literature, see: al-Rashida *et al.* (2013 ▶). For a related structure, see: Ishikawa & Motohashi (2013 ▶). For halogen bonding, see: Auffinger *et al.* (2004 ▶); Metrangolo *et al.* (2005 ▶); Wilcken *et al.* (2013 ▶); Sirimulla *et al.* (2013 ▶).
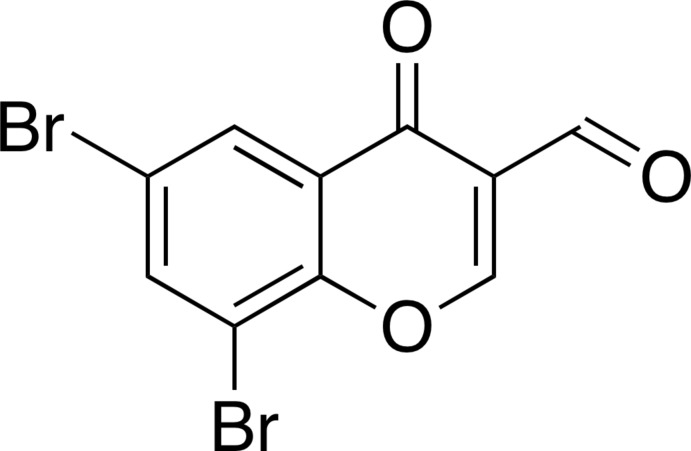


## Experimental   

### 

#### Crystal data  

C_10_H_4_Br_2_O_3_
*M* *_r_* = 331.95
Monoclinic, 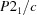

*a* = 11.910 (4) Å
*b* = 3.8500 (12) Å
*c* = 20.817 (6) Å
β = 95.69 (3)°
*V* = 949.8 (5) Å^3^
*Z* = 4
Mo *K*α radiation
μ = 8.54 mm^−1^
*T* = 100 K
0.42 × 0.25 × 0.23 mm

#### Data collection  

Rigaku AFC-7R diffractometer
Absorption correction: ψ scan (North *et al.*, 1968 ▶) *T*_min_ = 0.086, *T*_max_ = 0.140
2871 measured reflections
2163 independent reflections
1937 reflections with *F* ^2^ > 2σ(*F* ^2^)
*R*_int_ = 0.010
3 standard reflections every 150 reflections intensity decay: −0.1%

#### Refinement  

*R*[*F* ^2^ > 2σ(*F* ^2^)] = 0.019
*wR*(*F* ^2^) = 0.045
*S* = 1.06
2163 reflections
136 parameters
H-atom parameters constrained
Δρ_max_ = 0.37 e Å^−3^
Δρ_min_ = −0.46 e Å^−3^

### 

Data collection: *WinAFC* (Rigaku, 1999 ▶); cell refinement: *WinAFC*; data reduction: *WinAFC*; program(s) used to solve structure: *SIR92* (Altomare *et al.*, 1994 ▶); program(s) used to refine structure: *SHELXL97* (Sheldrick, 2008 ▶); molecular graphics: *CrystalStructure* (Rigaku, 2010 ▶); software used to prepare material for publication: *CrystalStructure*.

## Supplementary Material

Crystal structure: contains datablock(s) General, I. DOI: 10.1107/S1600536814005327/rn2123sup1.cif

Structure factors: contains datablock(s) I. DOI: 10.1107/S1600536814005327/rn2123Isup2.hkl

Supporting information file. DOI: 10.1107/S1600536814005327/rn2123Isup3.cml

CCDC reference: 990678

Additional supporting information:  crystallographic information; 3D view; checkCIF report

## Figures and Tables

**Fig. 1.**
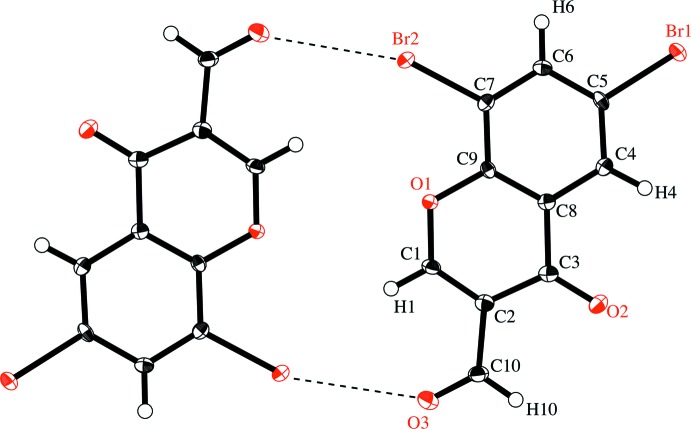
The molecular structure of the title compound, with displacement ellipsoids drawn at the 50% probability level. Hydrogen atoms are shown as small spheres of arbitrary radius. The intermolecular interaction of the title compound is represented as dashed lines for Br···O.

## supplementary crystallographic information

### 1. Comment

The title compound shows tumor cell-cytotoxic, anti-HIV, anti-*Helicobacter pylori*, and urease inhibitory activities (Kawase *et al.* 2007). In addition, it is used as a starting material for the synthesis of alkaline phosphatase inhibitors (al-Rashida *et al.* 2013).

The atoms of 6,8-dibromo-chromone ring in the title compound is essentially coplanar, and the largest deviations is 0.1109 (3) Å for Br2. The formyl group is slightly twisted with respect to the attached ring [C1–C2–C10–O3 = 11.5 (4)° and C3–C2–C10–O3 = -168.9 (3)°].

In the crystal, the molecules are linked to each other through intermolecular interactions of the Br2 atom with the O3 atom of the formyl group [Br2···O3; 3.118 (2) Å, C7–Br2···O3^i^ = 162.37 (8)°, Br2···O^i^–C10^i^ = 140.20 (15)° (i): -*x* + 2, -*y* + 1, -*z* + 1], as shown in Fig. 1. The short contact and the geometry of the Br···O interactions come within the range of halogen bonding (Auffinger *et al.* 2004). The similar geometry is found in the crystal structure of 6,8-dichloro-4-oxochromene-3-carbaldehyde (Ishikawa *et al.* 2013).

Halogen bonds have been found to occur in organic, inorganic, and biological systems, and have recently attracted much attention in medicinal chemistry, chemical biology, and supramolecular chemistry (Auffinger *et al.* 2004, Metrangolo *et al.* 2005, Wilcken *et al.* 2013, Sirimulla *et al.* 2013). Our analysis suggests that the strong inhibitory activity of the title compound against urease might be attributable to the halogen bond observed in the crystal, because 3-formylchromones without any halogen atom at the 8-position in the literature do not show the urease inhibitory activity (Kawase *et al.* 2007).

### 2. Experimental

Single crystals suitable for X-ray diffraction were obtained by slow evaporation of a 2-butanone solution of commercially available title compound at room temperature.

### 3. Refinement

The C(sp^2^)-bound hydrogen atoms were placed in geometrical positions [C—H 0.95 Å, *U*_iso_(H) = 1.2*U*_eq_(C)], and refined using a riding model.

### Crystal data

**Table:**

C_10_H_4_Br_2_O_3_	*F*(000) = 632.00
*M**_r_* = 331.95	*D*_x_ = 2.321 Mg m^−^^3^
Monoclinic, *P*2_1_/*c*	Mo *K*α radiation, λ = 0.71069 Å
Hall symbol: -P 2ybc	Cell parameters from 25 reflections
*a* = 11.910 (4) Å	θ = 15.0–17.3°
*b* = 3.8500 (12) Å	µ = 8.54 mm^−^^1^
*c* = 20.817 (6) Å	*T* = 100 K
β = 95.69 (3)°	Block, colorless
*V* = 949.8 (5) Å^3^	0.42 × 0.25 × 0.23 mm
*Z* = 4	

### Data collection

**Table:**

Rigaku AFC-7R diffractometer	*R*_int_ = 0.010
ω scans	θ_max_ = 27.5°
Absorption correction: ψ scan (North *et al.*, 1968)	*h* = −15→15
*T*_min_ = 0.086, *T*_max_ = 0.140	*k* = −2→4
2871 measured reflections	*l* = −14→26
2163 independent reflections	3 standard reflections every 150 reflections
1937 reflections with *F*^2^ > 2σ(*F*^2^)	intensity decay: −0.1%

### Refinement

**Table:**

Refinement on *F*^2^	Secondary atom site location: difference Fourier map
*R*[*F*^2^ > 2σ(*F*^2^)] = 0.019	Hydrogen site location: inferred from neighbouring sites
*wR*(*F*^2^) = 0.045	H-atom parameters constrained
*S* = 1.06	*w* = 1/[σ^2^(*F*_o_^2^) + (0.0209*P*)^2^ + 0.9331*P*] where *P* = (*F*_o_^2^ + 2*F*_c_^2^)/3
2163 reflections	(Δ/σ)_max_ = 0.002
136 parameters	Δρ_max_ = 0.37 e Å^−^^3^
0 restraints	Δρ_min_ = −0.46 e Å^−^^3^
Primary atom site location: structure-invariant direct methods	

### Special details

**Table:**

Refinement. Refinement was performed using all reflections. The weighted *R*-factor (*wR*) and goodness of fit (*S*) are based on *F*^2^. *R*-factor (gt) are based on *F*. The threshold expression of *F*^2^ > 2.0 σ(*F*^2^) is used only for calculating *R*-factor (gt).

### Fractional atomic coordinates and isotropic or equivalent isotropic displacement parameters (Å^2^)

**Table:**

	*x*	*y*	*z*	*U*_iso_*/*U*_eq_	
Br1	0.573035 (18)	0.93086 (6)	0.699129 (10)	0.01429 (7)	
Br2	0.985432 (18)	0.27016 (6)	0.660364 (10)	0.01402 (6)	
O1	0.88801 (13)	0.2982 (5)	0.52224 (7)	0.0135 (4)	
O2	0.59071 (14)	0.7059 (5)	0.43970 (8)	0.0183 (4)	
O3	0.78264 (15)	0.1122 (6)	0.32871 (8)	0.0230 (4)	
C1	0.85463 (19)	0.2641 (7)	0.45870 (10)	0.0137 (5)	
C2	0.75650 (19)	0.3792 (7)	0.42904 (10)	0.0138 (5)	
C3	0.67683 (19)	0.5722 (7)	0.46469 (10)	0.0132 (5)	
C4	0.63812 (18)	0.7387 (6)	0.57688 (10)	0.0125 (5)	
C5	0.67063 (18)	0.7382 (7)	0.64209 (10)	0.0122 (5)	
C6	0.77314 (18)	0.5989 (7)	0.66820 (10)	0.0129 (5)	
C7	0.84520 (18)	0.4570 (7)	0.62724 (10)	0.0123 (5)	
C8	0.71085 (18)	0.5882 (7)	0.53541 (10)	0.0119 (5)	
C9	0.81363 (18)	0.4500 (6)	0.56088 (10)	0.0111 (5)	
C10	0.7291 (2)	0.3114 (7)	0.35894 (11)	0.0173 (5)	
H1	0.9046	0.1492	0.4329	0.0165*	
H4	0.5682	0.8381	0.5602	0.0149*	
H6	0.7932	0.6016	0.7135	0.0155*	
H10	0.6668	0.4296	0.3368	0.0208*	

### Atomic displacement parameters (Å^2^)

**Table:**

	*U*^11^	*U*^22^	*U*^33^	*U*^12^	*U*^13^	*U*^23^
Br1	0.01503 (11)	0.01571 (12)	0.01299 (11)	0.00054 (9)	0.00562 (8)	−0.00072 (9)
Br2	0.01206 (11)	0.01685 (12)	0.01260 (11)	0.00133 (9)	−0.00153 (8)	0.00018 (9)
O1	0.0109 (8)	0.0193 (9)	0.0102 (7)	0.0017 (7)	0.0008 (6)	−0.0015 (7)
O2	0.0151 (8)	0.0233 (10)	0.0159 (8)	0.0032 (8)	−0.0015 (7)	0.0023 (8)
O3	0.0206 (9)	0.0327 (11)	0.0157 (8)	0.0031 (9)	0.0015 (7)	−0.0066 (8)
C1	0.0151 (11)	0.0162 (12)	0.0099 (10)	−0.0017 (10)	0.0013 (8)	−0.0029 (9)
C2	0.0142 (11)	0.0145 (12)	0.0128 (11)	−0.0025 (10)	0.0013 (9)	−0.0005 (9)
C3	0.0142 (11)	0.0137 (12)	0.0117 (10)	−0.0038 (10)	0.0013 (8)	0.0008 (9)
C4	0.0107 (10)	0.0117 (12)	0.0148 (11)	−0.0015 (9)	0.0003 (8)	0.0009 (9)
C5	0.0121 (11)	0.0114 (12)	0.0141 (10)	−0.0025 (10)	0.0072 (8)	−0.0014 (9)
C6	0.0150 (11)	0.0135 (12)	0.0103 (10)	−0.0022 (10)	0.0013 (8)	0.0006 (9)
C7	0.0109 (10)	0.0123 (12)	0.0136 (11)	−0.0007 (9)	−0.0004 (8)	0.0012 (9)
C8	0.0114 (10)	0.0125 (12)	0.0119 (10)	−0.0029 (10)	0.0017 (8)	0.0012 (9)
C9	0.0108 (10)	0.0110 (11)	0.0122 (10)	−0.0008 (9)	0.0041 (8)	0.0002 (9)
C10	0.0157 (11)	0.0241 (14)	0.0117 (11)	−0.0008 (11)	−0.0006 (9)	−0.0006 (10)

### Geometric parameters (Å, º)

**Table:**

Br1—C5	1.893 (3)	C4—C5	1.374 (3)
Br2—C7	1.885 (3)	C4—C8	1.407 (4)
O1—C1	1.349 (3)	C5—C6	1.394 (3)
O1—C9	1.384 (3)	C6—C7	1.381 (4)
O2—C3	1.217 (3)	C7—C9	1.395 (3)
O3—C10	1.213 (4)	C8—C9	1.391 (3)
C1—C2	1.342 (3)	C1—H1	0.950
C2—C3	1.464 (4)	C4—H4	0.950
C2—C10	1.486 (3)	C6—H6	0.950
C3—C8	1.489 (3)	C10—H10	0.950
			
Br2···O1	2.9940 (17)	C10···O2^v^	3.395 (4)
O1···C3	2.877 (3)	C10···O3^iii^	3.223 (4)
O2···C1	3.561 (3)	Br1···H4	2.9089
O2···C4	2.858 (3)	Br1···H6	2.9009
O2···C10	2.898 (4)	Br2···H6	2.9323
O3···C1	2.818 (3)	O2···H4	2.5993
C1···C7	3.599 (4)	O2···H10	2.6314
C1···C8	2.753 (4)	O3···H1	2.4918
C2···C9	2.775 (3)	C1···H10	3.2748
C4···C7	2.801 (3)	C3···H1	3.2875
C5···C9	2.750 (4)	C3···H4	2.6802
C6···C8	2.790 (3)	C3···H10	2.7091
Br1···Br1^i^	3.4567 (8)	C4···H6	3.2769
Br1···Br1^ii^	3.4567 (8)	C6···H4	3.2818
Br1···C5^iii^	3.563 (3)	C9···H1	3.1918
Br2···O3^iv^	3.118 (2)	C9···H4	3.2814
Br2···C7^v^	3.583 (3)	C9···H6	3.2634
O1···O1^vi^	3.298 (3)	C10···H1	2.5478
O1···C1^vi^	3.486 (3)	H1···H10	3.4736
O1···C8^v^	3.481 (3)	Br1···H10^vii^	3.1969
O1···C9^v^	3.498 (3)	Br1···H10^xi^	3.0191
O2···C2^iii^	3.280 (3)	Br2···H1^iv^	2.9314
O2···C3^iii^	3.513 (4)	Br2···H1^vi^	3.3140
O2···C4^vii^	3.208 (3)	Br2···H6^xii^	3.5908
O2···C4^viii^	3.455 (3)	O1···H1^iv^	3.0794
O2···C10^iii^	3.395 (4)	O1···H1^vi^	3.3226
O3···Br2^iv^	3.118 (2)	O2···H4^vii^	2.8234
O3···C2^v^	3.543 (4)	O2···H4^viii^	2.5820
O3···C6^ix^	3.430 (3)	O3···H6^ix^	2.5500
O3···C10^v^	3.223 (4)	O3···H10^v^	2.9808
C1···O1^vi^	3.486 (3)	C1···H1^iii^	3.5101
C1···C3^v^	3.413 (4)	C2···H1^iii^	3.4467
C1···C8^v^	3.577 (4)	C3···H4^vii^	3.3142
C2···O2^v^	3.280 (3)	C4···H4^v^	3.5744
C2···O3^iii^	3.543 (4)	C8···H4^v^	3.4152
C2···C3^v^	3.353 (4)	C10···H6^ix^	3.5682
C3···O2^v^	3.513 (4)	C10···H10^v^	3.4959
C3···C1^iii^	3.413 (4)	H1···Br2^iv^	2.9314
C3···C2^iii^	3.353 (4)	H1···Br2^vi^	3.3140
C4···O2^vii^	3.208 (3)	H1···O1^iv^	3.0794
C4···O2^viii^	3.455 (3)	H1···O1^vi^	3.3226
C4···C8^iii^	3.513 (4)	H1···C1^v^	3.5101
C4···C9^iii^	3.481 (4)	H1···C2^v^	3.4467
C5···Br1^v^	3.563 (3)	H4···O2^vii^	2.8234
C5···C6^iii^	3.555 (4)	H4···O2^viii^	2.5820
C5···C7^iii^	3.493 (4)	H4···C3^vii^	3.3142
C6···O3^x^	3.430 (3)	H4···C4^iii^	3.5744
C6···C5^v^	3.555 (4)	H4···C8^iii^	3.4152
C6···C7^iii^	3.539 (4)	H4···H4^viii^	3.1095
C7···Br2^iii^	3.583 (3)	H6···Br2^xiii^	3.5908
C7···C5^v^	3.493 (4)	H6···O3^x^	2.5500
C7···C6^v^	3.539 (4)	H6···C10^x^	3.5682
C8···O1^iii^	3.481 (3)	H6···H10^xi^	3.5884
C8···C1^iii^	3.577 (4)	H10···Br1^vii^	3.1969
C8···C4^v^	3.513 (4)	H10···Br1^xiv^	3.0191
C8···C9^iii^	3.558 (4)	H10···O3^iii^	2.9808
C9···O1^iii^	3.498 (3)	H10···C10^iii^	3.4959
C9···C4^v^	3.481 (4)	H10···H6^xiv^	3.5884
C9···C8^v^	3.558 (4)		
			
C1—O1—C9	117.88 (17)	C3—C8—C4	119.99 (19)
O1—C1—C2	125.3 (3)	C3—C8—C9	120.3 (2)
C1—C2—C3	120.9 (2)	C4—C8—C9	119.73 (19)
C1—C2—C10	119.3 (3)	O1—C9—C7	117.29 (19)
C3—C2—C10	119.8 (2)	O1—C9—C8	122.01 (19)
O2—C3—C2	124.0 (2)	C7—C9—C8	120.7 (2)
O2—C3—C8	122.7 (3)	O3—C10—C2	123.0 (3)
C2—C3—C8	113.31 (19)	O1—C1—H1	117.367
C5—C4—C8	118.4 (2)	C2—C1—H1	117.366
Br1—C5—C4	119.25 (17)	C5—C4—H4	120.820
Br1—C5—C6	118.34 (16)	C8—C4—H4	120.803
C4—C5—C6	122.4 (2)	C5—C6—H6	120.481
C5—C6—C7	119.05 (19)	C7—C6—H6	120.472
Br2—C7—C6	120.49 (16)	O3—C10—H10	118.496
Br2—C7—C9	119.78 (17)	C2—C10—H10	118.482
C6—C7—C9	119.7 (2)		
			
C1—O1—C9—C7	174.68 (18)	C8—C4—C5—Br1	−178.77 (18)
C1—O1—C9—C8	−4.8 (3)	C8—C4—C5—C6	0.9 (4)
C9—O1—C1—C2	3.1 (4)	H4—C4—C5—Br1	1.2
C9—O1—C1—H1	−176.9	H4—C4—C5—C6	−179.1
O1—C1—C2—C3	2.7 (4)	H4—C4—C8—C3	−2.2
O1—C1—C2—C10	−177.74 (19)	H4—C4—C8—C9	178.8
H1—C1—C2—C3	−177.3	Br1—C5—C6—C7	179.91 (14)
H1—C1—C2—C10	2.3	Br1—C5—C6—H6	−0.1
C1—C2—C3—O2	174.2 (3)	C4—C5—C6—C7	0.3 (4)
C1—C2—C3—C8	−6.2 (4)	C4—C5—C6—H6	−179.8
C1—C2—C10—O3	11.5 (4)	C5—C6—C7—Br2	179.31 (18)
C1—C2—C10—H10	−168.5	C5—C6—C7—C9	−1.1 (4)
C3—C2—C10—O3	−168.9 (3)	H6—C6—C7—Br2	−0.7
C3—C2—C10—H10	11.1	H6—C6—C7—C9	178.9
C10—C2—C3—O2	−5.4 (4)	Br2—C7—C9—O1	0.9 (3)
C10—C2—C3—C8	174.23 (19)	Br2—C7—C9—C8	−179.62 (14)
O2—C3—C8—C4	5.1 (4)	C6—C7—C9—O1	−178.7 (2)
O2—C3—C8—C9	−175.9 (2)	C6—C7—C9—C8	0.8 (4)
C2—C3—C8—C4	−174.51 (19)	C3—C8—C9—O1	0.8 (4)
C2—C3—C8—C9	4.5 (3)	C3—C8—C9—C7	−178.61 (19)
C5—C4—C8—C3	177.82 (19)	C4—C8—C9—O1	179.8 (2)
C5—C4—C8—C9	−1.2 (4)	C4—C8—C9—C7	0.4 (4)

Symmetry codes: (i) −*x*+1, *y*−1/2, −*z*+3/2; (ii) −*x*+1, *y*+1/2, −*z*+3/2; (iii) *x*, *y*+1, *z*; (iv) −*x*+2, −*y*, −*z*+1; (v) *x*, *y*−1, *z*; (vi) −*x*+2, −*y*+1, −*z*+1; (vii) −*x*+1, −*y*+1, −*z*+1; (viii) −*x*+1, −*y*+2, −*z*+1; (ix) *x*, −*y*+1/2, *z*−1/2; (x) *x*, −*y*+1/2, *z*+1/2; (xi) *x*, −*y*+3/2, *z*+1/2; (xii) −*x*+2, *y*−1/2, −*z*+3/2; (xiii) −*x*+2, *y*+1/2, −*z*+3/2; (xiv) *x*, −*y*+3/2, *z*−1/2.
